# Source of Previous Treatment for Re-Treatment TB Cases Registered under the National TB Control Programme, India, 2010

**DOI:** 10.1371/journal.pone.0022061

**Published:** 2011-07-21

**Authors:** Kuldeep Singh Sachdeva, Srinath Satyanarayana, Puneet Kumar Dewan, Sreenivas Achuthan Nair, Raveendra Reddy, Debasish Kundu, Sarabjit Singh Chadha, Ajay Kumar Madhugiri Venkatachalaiah, Malik Parmar, Lakhbir Singh Chauhan

**Affiliations:** 1 Central TB Division, Directorate General of Health Services, Ministry of Health and Family Welfare, Government of India, New Delhi, India; 2 South East Asia Regional Office, International Union Against Tuberculosis and Lung Diseases (The Union), New Delhi, India; 3 Office of the World Health Organisation (WHO) Representative in India, WHO Country Office, New Delhi, India; McGill University, Canada

## Abstract

**Background:**

In 2009, nearly half (289,756) of global re-treatment TB notifications are from India; no nationally-representative data on the source of previous treatment was available to inform strategies for improvement of initial TB treatment outcome.

**Objectives:**

To assess the source of previous treatment for re-treatment TB patients registered under India's Revised National TB control Programme (RNTCP).

**Methodology:**

A nationally-representative cross sectional study was conducted in a sample of 36 randomly-selected districts. All consecutively registered retreatment TB patients during a defined 15-day period in these 36 districts were contacted and the information on the source of previous treatment sought.

**Results:**

Data was collected from all 1712 retreatment TB patients registered in the identified districts during the study period. The data includes information on 595 ‘relapse’ cases, 105 ‘failure’ cases, 437 ‘treatment after default (TAD)’ cases and 575 ‘re-treatment others’ cases. The source of most recent previous anti-tuberculosis therapy for 754 [44% (95% CI, 38.2%–49.9%)] of the re-treatment TB patients was from providers outside the TB control programme. A higher proportion of patients registered as TAD (64%) and ‘retreatment others’ (59%) were likely to be treated outside the National Programme, when compared to the proportion among ‘relapse’ (22%) or ‘failure’ (6%). Extrapolated to national registration, of the 292,972 re-treatment registrations in 2010, 128,907 patients would have been most recently treated outside the national programme.

**Conclusions:**

Nearly half of the re-treatment cases registered with the national programme were most recently treated outside the programme setting. Enhanced efforts towards extending treatment support and supervision to patients treated by private sector treatment providers are urgently required to improve the quality of treatment and reduce the numbers of patients with recurrent disease. In addition, reasons for the large number of recurrent TB cases from those already treated by the national programme require urgent detailed investigation.

## Introduction

The primary aim of the tuberculosis (TB) control programmes across the world is to reduce mortality and morbidity due to TB by interrupting the chain of TB transmission [1]. Re-treatment cases (TB Patients who have been previously treated with anti-TB drugs for at least a month), are a challenge to this primary aim of the TB control Programmes. When compared to new cases, re-treatment cases require longer and more complicated treatment, are more likely harbour & transmit drug-resistant *TB*, and are likely to have poor treatment outcomes, including increased risk of death [2], [3].

In 2009, 622,342 (10%) of the 6.12 million global total TB notifications million were re-treatment TB cases. India is estimated to have more than one-fifth of the global TB burden. India, however, disproportionately accounts for nearly half of re-treatment TB cases notified globally, with 289,756 notified re-treatment cases in 2009 [4]. The notification rate of re-treatment TB in India has slowly but steadily increased over the past decade, from 14 cases per 100,000 population in 2001 to 25 cases per 100,000 population in 2009 [5].

Re-treatment cases emerge mostly as a result of inadequate and improper treatment of the new-TB cases, but TB treatment is widely available in India from the public and private sector alike, with or without a prescription. Very little data has been reported on the source of previous treatment for re-treatment cases under programmatic conditions; only one small study from 2003 in Rajasthan, North India, reported more than 85% of the retreatment cases studied were previously treated in the private sector [6].

The knowledge on the proportion of patients previously treated in the non-RNTCP sector will assist the programme to interpret the implication of the increasing notification of re-treatment cases. It is unclear to what extent re-treatment cases notified represents a warning sign regarding long term treatment outcomes under RNTCP, or to what extent this represents the influence of treatment of TB patients by the private sector. Hence, we undertook nationally representative survey to assess the source of previous treatment for re-treatment TB patients registered under India's RNTCP.

## Methods

### Study setting

The Government of India's Revised National TB Control Programme (RNTCP), based on the internationally recommended Directly Observed Treatment Short-course (DOTS) strategy, was launched in 1997, expanded across the country in a phased manner and full nationwide coverage was achieved in March 2006. TB cases are diagnosed, categorized and treated with thrice-weekly intermittent treatment regimens as per the standard WHO treatment guidelines [3], [7]. Health care in India is provided by both public and huge non-public sector health facilities and TB patients are diagnosed and managed both in the public as well in the non-public health sector facilities [8]. Patients from the public sector are usually managed under the programmatic setting as specified under RNTCP guidelines and are part of the TB notification system in India. RNTCP has made major efforts to involve non-public health sector facilities, but many patients (unknown magnitude) are still managed outside the programmatic setting. Those in the private sector may or may not be managed as per programmatic guidelines and are outside the TB notification system of the country. The present study deals with the patients notified under RNTCP.

### Study design, sample size, sampling and study population

We designed a nationally-representative cross-sectional study, with cluster sampling, districts being the primary sampling units (Clusters) and 100% enrolment of re-treatment patients in selected districts for a fixed enrolment window. In the absence of reliable estimates, we assumed that 50% of the re-treatment cases would have previously been treated under RNTCP. A sample size of 880 patients was selected to estimate a 50% prevalence with a precision of ±5%, considering a 10% non-response rate and with a design effect of 2 to account for cluster sampling. There were 650 districts in the country with varying population sizes and numbers of re-treatment TB patients registered. We selected 36 districts by population proportionate to size sampling methodology. Based on notification rates, a 15 day enrolment period was considered sufficient to achieve the desired sample size. The study population were all TB cases that were registered as re-treatment TB cases during the study period.

### Definitions of study variables

As routine programme practice, after the diagnosis of TB, every TB patient is asked about the history of TB treatment by the treating medical officer which includes reviewing previous medical records and asking if s/he has ever taken treatment for TB, injections for more than one or two weeks (streptomycin is likely), or taken a medicine, which turned the urine orange–red (rifampicin is likely). In addition to this, patients are also informed that hiding her/his past treatment for TB, will result in wrong treatment which can lead to failure of treatment and even death. A retreatment TB patient under RNTCP is defined as a TB patient who has taken anti-tuberculosis drugs for more than one month from any source. If previously treated, the details on the year of treatment, duration of treatment, drugs taken and source of treatment (RNTCP/non-RNTCP) are recorded on the treatment cards of the TB patients [9].

Re-treatment TB patients are further classified by the treating medical officer at the time of treatment initiation into one of 4 registration groups: ‘relapse’ (a TB patient who was declared cured or treatment completed by a physician in the past, but who reports back to the health service and is now found to be sputum smear-positive), ‘treatment after failure’ (a TB patient who is smear-positive at 5 months or more after starting treatment and also a initial smear negative TB patient who turns out to be smear positive is also considered as a failure), ‘treatment after default (TAD)’, (a TB patient who received anti-tuberculosis treatment for one month or more from any source and returns to treatment after having defaulted, i.e., not taken anti-TB drugs consecutively for two months or more, and is found to be sputum smear-positive) or ‘re-treatment others’ (retreatment TB patients who do not fit into the above mentioned types).

All types of re-treatment TB patients are treated with a thrice-weekly fully-intermittent re-treatment regimen of 8 to 9 months duration under direct observation. The first three months of intensive phase consists of approximately two months (24 doses) with 5 drugs [Isonaizid (H), rifampicin (R), pyrazinamide (Z), ethambutol (E) and streptomycin (S)] followed by one month (12 doses) of four drugs (H,R,Z,E). If the patient is sputum smear positive at the end of 3 months (36 doses) , the intensive phase is extended for another month (12 doses) with H,R,Z,E. Irrespective of the sputum smear status at the end of extended intensive phase, the continuation phase with three drugs (H,R,E) is started and continued for 5 months (66 doses). All patients initiated on treatment are registered in a TB register maintained by a paramedical worker (Senior TB Supervisor).

### Data collection

The data was collected by a paramedical Senior TB Supervisors by interviewing the re-treatment TB patients at the time of registration and cross verified by the local Medical Officers and programme managers. In addition, Medical consultants of the WHO-RNTCP technical assistance project cross verified by interviewing 10% of the data collected by the district programme staff. Given the large, decentralized nature of data collection, and based on the experiences during pre-testing of the study methodology and the data collection sheet, the variables on which the data was collected was kept fairly simple. The data was collected on the most recent source of previous treatment; whether it was ‘under RNTCP’ or ‘non-RNTCP’, the age, sex, type of re-treatment TB. Patients were considered to be previously treated under RNTCP, if they could recollect that they were treated from a ‘patient wise box’ by visiting a DOT Provider thrice weekly or if they possessed a ‘patient identity card’ provided by RNTCP or if the ‘treatment supervisors’ were able to identify a TB registration number under which these were previously treated by cross-verifying the program records or by reviewing the medical records. In the absence of any of this evidence of drug intake from RNTCP, patients were considered to be previously treated by ‘non-RNTCP sources’.

### Ethical Considerations

The protocol was reviewed and approved by Central TB Division, Ministry of Health and Family Welfare, Government of India. As per programme guidelines, the history of previous treatment needs to be elicited from every patient and recorded in a systematic manner on the treatment cards. This study used the same procedure to collect the data from the patients. Since the study collected the data from an established practice as per the programme guidelines, within the framework of routine care, individual patient consent was deemed unnecessary by The Central TB Division.

### Data entry and analysis

Data collected from the field by the investigators were entered into a pre-structured format in Microsoft Excel, cross verified by the District TB Officers and then sent to Central TB Division. At Central TB Division, data from individual districts was compiled from all the 36 districts. Data was analysed using Epi Info (TM) Version 3.5.1. Complex sample analysis was done with districts as the primary sampling units to account from cluster sampling methodology. Variables are summarized by proportions and 95% confidence intervals (95% CI). Differences between sub-groups are measured by chi-square tests.

## Results

During a 15 day period 16^th^ May–31^st^ May, 2010, in the 36 selected districts a total of 1712 re-treatment patients were registered, and all 1712 were enrolled. For operational reasons one district collected data from all consecutively registered patients from 1^st^ August–15^th^ August, 2010. The Medical Consultants of the WHO-RNTCP technical assistance project re-visited 181 patients, cross verified the data collected and there was only one case in which as error was noticed in recording the source of previous treatment.

Seventy percent of the re-treatment cases were males (95% CI; 66.6%–73.4%), with 66% in the age-group of 25–54 years. Patients classified as registration type ‘relapse’ and ‘others’ represented two-thirds of the patients registered, one-fourth were TAD, and the remaining 6% were classified as ‘treatment after failure’ re-treatment registrations. The source of most recent previous anti-tuberculosis therapy for 56% (95% CI; 50.1%–61.8%) of the retreatment cases was from RNTCP and the remaining from non-RNTCP sources (Table 1). There were no age and sex differences between those previously treated under RNTCP and non-RNTCP sources. ‘Relapse’ and ‘failure’ cases are more likely to have been previously treated under RNTCP and ‘TAD’ and ‘re-treatment other’ are more likely to have been previously treated from Non-RNTCP sources (Table 2).

**Table 1:** Demographic characteristics and source of previous treatment of re-treatment TB patients registered under RNTCP in India, 2010 (n = 1712).

Variable	FrequencyN (%)	95% CI
**Age distribution**		
<15 years	34 (2.0)	1.3–2.7
15–24 yrs	287(16.8)	13.7–19.9
25–34 Yrs	395 (23.1)	20.8–25.4
35–44 yrs	398 (23.2)	21.2–25.3
45–54 Yrs	345 (20.2)	17.3–23.0
55–64 yrs	166(9.7)	7.6–11.8
≥65 yrs	87 (5.1)	3.6–6.5
**Sex**		
Female	514 (30.0)	26.6–33.4
Male	1198 (70.0)	66.6–73.4
**Type of TB**		
Relapse	595 (34.8)	27.7–41.8
Failure	105 (6.1)	4.6–7.7
TAD	437 (25.5)	20.7–30.3
Others	575 (33.6)	25.2–42.0
**Previous treatment source**		
Non RNTCP	754 (44.0)	38.2–49.9
RNTCP	958 (56.0)	50.1–61.8

**Table 2:** Association between demographic variables and type of TB with the history of previous source of treatment, India.

Variable	Non RNTCP		RNTCP		TOTAL	Chi square test P value
**Sex**	**n**	**%**	**n**	**%**		
Female	240	47%	274	53%	514	P = 0.1480
Male	514	43%	684	57%	1198	
**Age**						
<15 years	14	41%	20	59%	34	P = 0.3973
15–24 yrs	129	45%	158	55%	287	
25–34 Yrs	181	46%	214	54%	395	
35–44 yrs	165	41%	233	59%	398	
45–54 Yrs	141	41%	204	59%	345	
55–64 yrs	84	51%	82	49%	166	
>65 yrs	40	46%	47	54%	87	
**Type of TB**						
Relapse	133.0	22%	462	78%	595	Referent
Failure	6.0	6%	99	94%	105	0.00008
TAD	278.0	64%	159	36%	437	0.00000
Others	337.0	59%	238	41%	575	0.00000

## Discussion

Half of global TB re-treatment notifications are from India [4], and reasons for this disproportionate contribution require investigation. This is the first nationally representative operational research study on the source of previous treatment for re-treatment TB patients registered under the programme in India. Nearly half (44%) of the re-treatment TB patients registered under the RNTCP in India were previously treated from ‘non-RNTCP sources’. The study findings have the following implications on the TB control efforts in the country.

First, extrapolated to national registration, of the 292,972 re-treatment registrations in 2010, 128,907 (95% CI, 111,915–146,193) patients would have been most recently treated outside the national programme. This key finding provides a direct measure quantifying a part of the large number of TB cases diagnosed and managed outside the national TB programme. Tremendous efforts have been made by the programme to involve all types of health care providers in the country in an attempt to reach out to all the TB patients and substantial successes have been achieved with the involvement of large numbers of medical colleges, non-governmental organisation health facilities, and other public sector hospitals [5], [8], [10]. The study findings however, suggest that much more efforts are still needed.

Second, treating re-treatment TB patients is challenging due to high levels of default [11] and prevalence of MDR-TB [12] and only 70% of registered re-treatment TB cases are successfully treated under programmatic setting. Ensuring early diagnosis of new TB patients and its prompt treatment' under programmatic setting is the epidemiological basis of TB control strategy and the only known cost effective measure to break the chain of TB transmission for the control of tuberculosis. This is the basis of the WHO recommended DOTS strategy and also the present global strategy to stop TB [1]. The responsibility for participating in organised TB control efforts also rests with all health care providers who manage TB patients as per the International Standards of TB Care [13]. All stakeholders associated with Tuberculosis control must realise the importance of preventing ‘new’ cases from becoming ‘retreatment’ TB cases.

Third, more than half of the patients were most recently previously treated from within the RNTCP. This raises questions about the long term efficacy of short-course chemotherapy in the programme setting, and the accuracy of smear microscopy at the end of treatment in identifying patients with residual disease below the threshold of microscopy detection. In addition, ∼2% of the sputum smear positive TB patients are declared as ‘treatment completed’ without having undergone end of treatment follow-up sputum smear examination [5]. In perspective, a huge cohort of more than 12 million TB patients has been treated under the programme since its implementation. This study provides no insight into the rate of long-term TB treatment success or failure of the treatment regimen and appropriateness of the programmatic processes adopted by RNTCP in treating patients and in declaring treatment outcomes. To address this concern, the programme should study long term treatment outcomes of TB patients treated with fully intermittent short-course chemotherapy under programmatic conditions.

Fourth, retreatment tuberculosis patients classified as ‘TAD’ and ‘retreatment-others’ are more likely to have been treated outside the programmatic setting. While ‘TAD’ cases are sputum smear positive pulmonary TB cases, ‘re-treatment others’ could be sputum smear negative or extra-pulmonary TB cases. A previous study in India had showed that a vast majority (84%) of the ‘re-treatment others’ were sputum smear negative pulmonary TB cases with relatively better treatment outcomes than sputum smear positive re-treatment TB cases [14].

Finally, the influence of known risk factors for poor long term treatment outcomes will have to be considered; as the prevalence of irregular drug intake at the time of tuberculosis treatment, tobacco usage, diabetes, HIV infection and malnutrition, and other risk factors is likely to affect long term treatment efficacy rates [15]–[18]. Measure to address these known risk factors, needs to be incorporated within the programme framework.

### Limitations

As in all research studies, there a few limitations in the present study that needs to be considered while accepting the data. First, this study is from patients were registered for treatment under RNTCP. We believe that a large number of TB patients (unknown magnitude) in India are treated outside the programmatic setting [19]. This study only represents patients who registered for treatment under RNTCP and not outside RNTCP. The choice of health provider and source of re-treatment chosen by the patients may have affected the profile of re-treatment TB patients registered under the programme. There is no information on the magnitude of TB patients who prefer to be treated from the same source or a different source. Second, the past treatment profile of re-treatment TB patients is affected by the number and different types of health care providers offering TB treatment services in any given area and the reach of RNTCP to these different types of the health care providers. Third, we chose a 15 day period for data collection based on operational feasibility and not randomly. If there are seasonal changes in seeking health care from RNTCP, then this profile may be different. Lastly, the study was done in a programmatic setting and all the study investigators are linked with the programme. They may have their own inherent biases when it comes to deciding on the source of previous treatment for any patient. In order to minimise classification and recall bias, limited information on only the most recent source of previous treatment was collected. The data was cross-verified by the local programme mangers, and the additional sample re-verified by WHO-RNTCP medical consultants suggested high accuracy in initial classification. Hence misclassification of results did not likely affect our key findings.

### Conclusions

Nearly half of the re-treatment TB cases registered under RNTCP for treatment were previously treated from non-RNTCP sources. There are no age and sex differentials in the source of most recent previous treatment. Among retreatment-TB types, ‘relapses’ and ‘failures’ are more likely to have been treated under RNTCP and ‘treatment after default’ and ‘re-treatment others’ are likely to have been previously treated from non-RNTCP sources.

### Recommendations

This study can form the starting point for a useful conversation to address the challenges of re-treatment TB patients in India, which has a substantial implication for the global TB control. Enhanced efforts towards extending treatment support and supervision to patients treated by private sector treatment providers are urgently required to improve the quality of treatment and reduce the numbers of patients with recurrent disease. In addition, reasons for the large number of recurrent TB cases from those already treated by the national programme require urgent detailed investigation.

## References

[1] Stop TB Partnership and World Health Organization Geneva (2006).

[2] Rusen ID (2009). Tuberculosis retreatment: a topic whose time has come.. Int J Tuberc Lung Dis.

[3] World Health Organisation, Geneva (2010).

[4] World Health Organisation, Geneva (2010).

[5] Central Tuberculosis Division (2010). Tuberculosis India 2010..

[6] Sisodia RS, Wares DF, Sahu S, Chauhan LS, Zignol M (2006). Source of retreatment cases under the revised national TB control programme in Rajasthan, India, 2003.. Int J Tuberc Lung Dis.

[7] Central Tuberculosis Division (2005). Technical and Operational Guidelines for Tuberculosis Control, Revised National Tuberculosis Control Programme..

[8] Lal SS, Sahu S, Wares F, Lonnroth K, Chauhan LS (2011). Intensified scale-up of public-private mix: a systems approach to tuberculosis care and control in India.. Int J Tuberc Lung Dis.

[9] Central TB Division (2008). Revised Recording and reporting formats (addendum to the existing RNTCP training modules), Directorate General of Health Services, Ministry of Health and Family Welfare, Government of India.. http://www.tbcindia.org/pdfs/Addendum%20%20Revised%20recording%20and%20reporting%20formats%20120308%20Final.pdf.

[10] Dewan PK, Lal SS, Lonnroth K, Wares F, Uplekar M (2006). Improving tuberculosis control through public-private collaboration in India: literature review.. BMJ.

[11] Jha UM, Satyanarayana S, Dewan PK, Chadha S, Wares F (2010). Risk factors for treatment default among re-treatment tuberculosis patients in India, 2006.. PLoS One.

[12] Ramachandran R, Nalini S, Chandrasekar V, Dave PV, Sanghvi AS (2009). Surveillance of drug-resistant tuberculosis in the state of Gujarat, India.. Int J Tuberc Lung Dis.

[13] Fair E, Hopewell PC, Pai M (2007). International Standards for Tuberculosis Care: revisiting the cornerstones of tuberculosis care and control.. Expert Rev Anti Infect Ther.

[14] Srinath S, Sharath B, Santosha K, Chadha SS, Roopa S (2011). Tuberculosis ‘retreatment others’: profile and treatment outcomes in the state of Andhra Pradesh, India.. Int J Tuberc Lung Dis.

[15] Thomas A, Gopi PG, Santha T, Chandrasekaran V, Subramani R (2005). Predictors of relapse among pulmonary tuberculosis patients treated in a DOTS programme in South India.. Int J Tuberc Lung Dis.

[16] Vijay S, Kumar P, Chauhan LS, Vollepore BH, Kizhakkethil UP (2010). Risk factors associated with default among new smear positive TB patients treated under DOTS in India.. PLoS One.

[17] Santha T, Garg R, Frieden TR, Chandrasekaran V, Subramani R (2002). Risk factors associated with default, failure and death among tuberculosis patients treated in a DOTS programme in Tiruvallur District, South India, 2000.. Int J Tuberc Lung Dis.

[18] Narayanan S, Swaminathan S, Supply P, Shanmugam S, Narendran G (2010). Impact of HIV infection on the recurrence of tuberculosis in South India.. J Infect Dis.

[19] Wells WA, Ge CF, Patel N, Oh T, Gardiner E (2011). Size and Usage Patterns of Private TB Drug Markets in the High Burden Countries.. PLoS One.

